# Epicardial fat volume is related to the degree of cardiac allograft vasculopathy

**DOI:** 10.1007/s00330-022-09029-2

**Published:** 2022-08-20

**Authors:** Stefan Roest, Ricardo P. J. Budde, Jasper J. Brugts, Jan von der Thüsen, Theo van Walsum, Yannick J. H. J. Taverne, Felix Zijlstra, Daniel Bos, Olivier C. Manintveld

**Affiliations:** 1 Department of Cardiology, Thorax Center, Erasmus MC, University Medical Center Rotterdam, Rotterdam, The Netherlands; 2 Erasmus MC Transplant Institute, Erasmus MC, University Medical Center Rotterdam, Rotterdam, The Netherlands; 3 Department of Radiology and Nuclear Medicine, Erasmus MC, University Medical Center Rotterdam, Rotterdam, The Netherlands; 4 Department of Pathology, Erasmus MC, University Medical Center Rotterdam, Rotterdam, The Netherlands; 5 Department of Cardiothoracic Surgery, Thorax Center, Erasmus MC, University Medical Center Rotterdam, Rotterdam, The Netherlands; 6 Department of Epidemiology, Erasmus MC, University Medical Center Rotterdam, Rotterdam, The Netherlands; 7 Department of Cardiology, Thorax Center, Erasmus MC, University Medical Center Rotterdam, Room RG-431, Doctor Molewaterplein 40, 3015 GD, Rotterdam, The Netherlands

**Keywords:** Cardiac allograft vasculopathy, Coronary computed tomography, Coronary artery disease, Epicardial fat, Heart transplantation

## Abstract

**Objectives:**

Increasing evidence suggests a role for epicardial fat in the development of coronary artery disease in the general population. Heart transplantation patients are at increased risk of developing a specific form of coronary artery disease, cardiac allograft vasculopathy (CAV), which has far-reaching consequences in terms of morbidity and mortality. Until now, the role of epicardial fat volume (EFV) in the development of CAV remains unknown. Hence, we investigated the relationship between EFV and CAV as well as the influence of donor/recipient sex on EFV.

**Methods:**

Adult heart transplant patients who underwent coronary computed tomography angiography (CCTA) for CAV screening who were four or more years post-HT were included. Using the CT examinations, we quantified the EFV and the degree of CAV. Ordinal and linear regression models were used to assess the association of EFV with CAV.

**Results:**

In total, 149 (median age 44.5 years, 36% women) patients were included. The median time between HT and the CT scan was 11.0 (7.3–16.1) years. CAV grade 0, 1, 2 and 3 were seen in 85 (57%), 32 (22%), 14 (9%), and 18 (12%) patients, respectively. The median EFV was 208.4 (128.9–276.0) mL. Larger EFV were related to higher degrees of CAV (median of 164.7 to 290.6 mL for CAV grade 0 and 3, respectively, OR 5.23 (2.47–11.06), *p* < 0.001). Male recipients had significantly more EFV than female recipients irrespective of the donor sex (232.7 mL vs. 147.2 mL respectively, *p* < 0.001). Determinants for EFV were recipient sex, number of rejections, donor age, time between HT and CT scan, recipient BMI, and diabetes mellitus.

**Conclusions:**

EFV was associated with higher degrees of CAV. The recipient sex influenced the EFV more than the donor sex.

**Key Points:**

*• Patients after heart transplantation have a high amount of epicardial fat while larger amounts of epicardial fat are related to higher grades of cardiac allograft vasculopathy.*

*• Determinants of higher epicardial fat volume included recipient sex, number of rejections, donor age, time between HT and CT scan, recipient BMI, and diabetes mellitus.*

*• Longitudinal studies are needed to further disentangle the role of epicardial fat in the development and progression of cardiac allograft vasculopathy.*

**Graphical abstract:**

**Figure:**
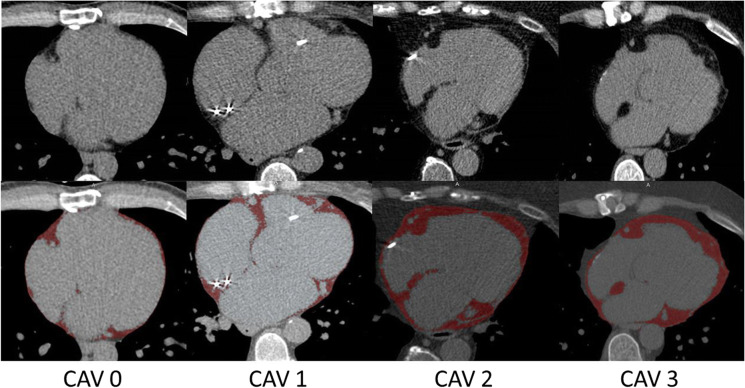
Demonstration of four patients (from CAV grade 0 to CAV grade 3) in whom epicardial fat volume was determined. In red, the voxels identified as epicardial fat.

**Supplementary Information:**

The online version contains supplementary material available at 10.1007/s00330-022-09029-2.

## Introduction

Cardiac allograft vasculopathy (CAV) is a major complication after heart transplantation (HT), with a 10-year incidence of up to 50% [1]. CAV has far-reaching consequences and thereby exerts a huge contribution to morbidity and mortality [1]. CAV is an accelerated form of coronary artery disease, which is characterized by intima hyperplasia along the whole length of the coronaries [2–4]. The exact pathophysiology of CAV remains largely unknown; however, it is acknowledged that it is influenced by a combination of both immunological and non-immunological risk factors [2, 5].

Given the serious consequences of CAV and the fact that HT patients do not experience symptoms following ischemia due to the denervation of the heart, annual or biannual screening for CAV is advocated in the International Society for Heart and Lung Transplantation (ISHLT) guidelines [2]. Despite substantial drawbacks, such as high radiation dose and peri-procedural complications, the screening procedure is advised to be done with coronary angiography [2]. To overcome these drawbacks, at our institution, we successfully implemented coronary computed tomography angiographies (CCTAs) for routine screening of CAV in 2018 [6, 7]. Because CT scans contain much more information, beyond only luminal stenosis, which is assessed with invasive coronary angiography, CAV can be detected in an earlier stage of the disease [8].

In this light, specific interest has risen around the amount of epicardial fat, a layer of fat surrounding the coronaries that is situated between the myocardium and visceral pericardium [9]. Several studies have demonstrated that in the general population, people with larger amounts of epicardial fat have an increased risk for coronary artery disease (CAD) [10–16]. The effects of epicardial fat volume (EFV) even reach beyond CAD and are also linked to atrial fibrillation, heart failure with preserved ejection fraction, and larger amounts of atherosclerosis [13, 17, 18]. Specifically in HT recipients, EFV may play an important role in the development of CAV, given its direct contact with the coronary arteries, and its excretion of pro-inflammatory factors [19, 20]. Currently, no studies have been performed to investigate the link between EFV and CAV. The primary aim of this study was to investigate the relationship between EFV and CAV. Furthermore, we investigated determinants of EFV in HT recipients and whether the donor and/or recipient sex has an influence on EFV.

## Material and methods

All adult patients that were seen at our outpatient clinic and were more than 4 years post-HT who underwent a CT for annual/biannual CAV screening were included in the study. Patients with an impaired renal function (estimated glomerular filtration rate < 45 mL/min/1.73 m^2^ before March 15, 2018, or < 30 mL/min/1.73 m^2^ after March 15, 2018, due to a change in the contrast nephropathy prevention protocol at our institution) or iodine allergy were excluded from CCTA [6]. All first CT scans performed between February 2018 and June 2020 were included. CT scans in which CAV scores or epicardial fat volumes could not be determined were excluded from the analysis. Patients who underwent simultaneous organ transplantation (i.e., heart-lung transplantation) were excluded. The study was approved by the Medical Ethical Review Committee (MEC-2017-421) and conformed with the Declaration of Helsinki.

### Assessment of epicardial fat

All scans were performed using the second- or third-generation dual-source CT scans (Somatom Definition Drive or Force, Siemens Healthineers) [6]. A scan consisted of a non-enhanced cardiac CT scan for coronary calcium scoring, followed by a CCTA. The non-enhanced CT was acquired at 120 kVp and reconstructed at 3-mm thick slices. The amount of epicardial fat was determined on the non-contrast enhanced scan. We used a validated custom-made algorithm that automatically delineated the pericardium [21]. Next, within the segmented pericardium, the EFV was determined by adding all voxels with Hounsfield unit (HU) thresholds between −200 and −30 and expressed in milliliters (mL) [21]. All regions smaller than 10 voxels were considered noise and not added to the total EFV (example in the graphical abstract) [21].

### Assessment of CAV

The presence and severity of CAV were evaluated on CCTA according to the ISHLT guidelines by an experienced cardiovascular radiologist with over 10 years’ experience [4, 6]. However, since the ISHLT guidelines on CAV are based on coronary angiography findings and not on CCTA findings, a diameter stenosis grade of 50% instead of 70% was deemed significant as advised by the Society for Cardiovascular Computed Tomography (SCCT) [22]. The exact definition of CAV and other relevant definitions are mentioned in the Supplemental Material.

### Statistical analyses

The EFV was natural log-transformed in order to correct for the right-skewed, non-normal distribution. Using ordinal logistic regression models, we investigated the association between EFV and CAV. In the first model, we added EFV unadjusted. In the second model, we adjusted for sex and age of the recipient. In order not to overfit the statistical model, we computed propensity scores for each patient that captured all potential confounders of the association of EFV with CAV. This propensity score was used as covariable in model three and included the following variables: sex and age of the recipient, time since heart transplantation, BMI recipient at the CT scan, diabetes, smoking, number of rejections, CMV infection post-heart transplant, and ischemia time. Since hypertension (83%) and cholesterol lowering medication (89%) were very common in the study population, these could not be included in the model. We adjusted for recipient age in an attempt to capture unknown confounding factors in the recipient that may influence the association of EFV with CAV. Given the considerable, statistically significant correlation with donor age (Spearman’s correlation: 0.59, *p* < 0.001), we did not include both ages (multicollinearity).

Next, using a linear regression model, we investigated determinants of EFV. First, a univariable analysis was performed including all potential determinants separately (listed below). Next, all variables that were associated with EFV with a *p* value ≤ 0.15 were simultaneously entered into a multivariable model. We specifically investigated associations of sex and age of the recipient, sex and age of the donor, number of rejections, time between HT and CCTA, prednisolone use, total cholesterol, low-density lipoprotein (LDL), BMI of the recipient, diabetes mellitus, and smoking post-HT with EFV. In order to investigate the effect of the sex of the recipient and the donor on the amount of epicardial fat, four groups were created (group 1, male recipient, male donor; group 2, male recipient, female donor; group 3, female recipient, male donor; group 4, female recipient, female donor). A Kruskal-Wallis test was performed to investigate the differences in median EFV per group. All statistical analyses were performed using IBM SPSS statistics 25 (IBM Corp.).

## Results

**Fig. 1:**
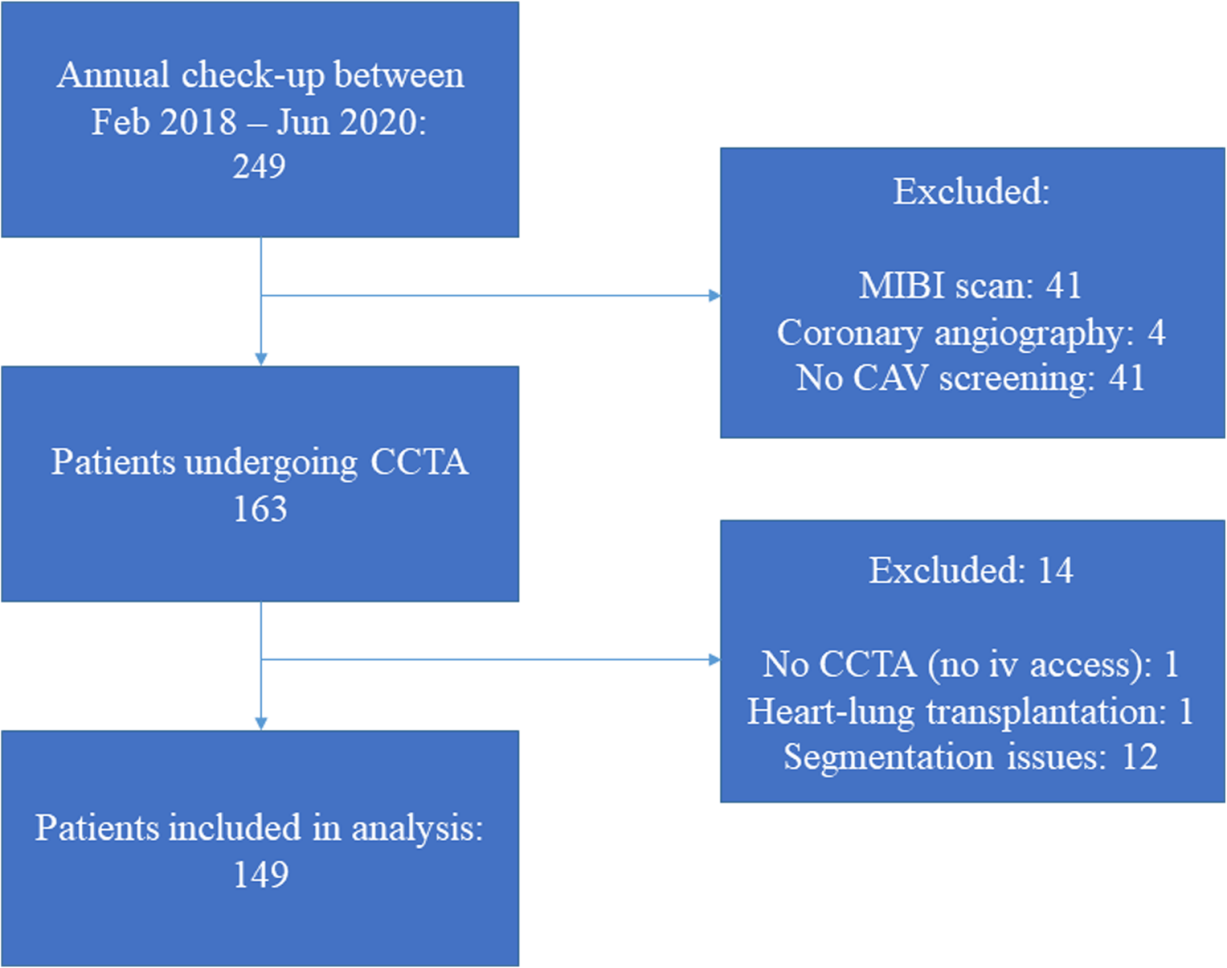
Flowchart demonstrating the in- and exclusion of patients in this study. Abbreviations: CAV, cardiac allograft vasculopathy; CCTA, coronary computed tomography angiography; iv, intravenous; MIBI, myocardial perfusion imaging

In total, 249 patients more than four years post-HT were seen at the outpatient clinic during the study period. After exclusion criteria, 149 were included in the analysis. In Fig. 1, a flowchart demonstrating the inclusion and exclusion of this study is demonstrated. Table 1 shows the characteristics of the study population (*n* = 149). The median age at the time of HT was 44.5 (28.0–53.6) years and 54 (36%) were female. The most frequent reason for HT was a non-ischemic cardiomyopathy (71%). Most of the donors were female (57%) and donors had a median age of 39.0 (21.0–48.5) years. The median time between HT and CT scan was 11.0 (7.3–16.1) years and patients had a median of 1 (0–2) rejections. CMV infections occurred in 30% of patients.

**Table 1:** Baseline characteristics of patients included in the study (*n* = 149)

Baseline characteristics	
Donor characteristics	
Age (years)	39.0 [21.0–48.5]
Female	85 (57)
BMI (kg/m^2^)	23.5 ± 3.6
Recipient characteristics	
Female	54 (36)
Caucasian	126 (85)
Age at HT (years)	44.5 [28.0–53.6]
Ischemic CMP	44 (30)
Non-ischemic CMP	105 (71)
LVAD	22 (15)
Diabetes pre-HT	7 (5)
Creatinine at HT (μmol/L)	111 ± 44
Surgical data	
Ischemic time (min)	184 [153–216]
Data at the time of CT scan	
Time since HT	11.0 [7.3–16.1]
BMI (kg/m^2^)	25.9 [23.5–28.8]
Number of rejections	1 [0–2]
CMV infection	44 (30)
Smoking post-HT	10 (7)
Diabetes mellitus	38 (26)
Hypertension	124 (83)
Immunosuppressive regimen	
Tacrolimus	128 (86)
Ciclosporin	20 (13)
Prednisolone	100 (67)

Continuous variables that are normally distributed are demonstrated as a mean ± standard deviation, non-normally distributed continuous variables as medians (25^th^–75^th^ percentile (Interquartile Range)). Categorical variables are demonstrated with absolute numbers (percentages)

Abbreviations: *BMI*, body mass index; *CMP*, cardiomyopathy; *CMV*, cytomegalovirus; *CT*, computed tomography; *HT*, heart transplantation; *LVAD*, left ventricular assist device

### Distribution of epicardial fat volume and prevalence of CAV

**Fig. 2:**
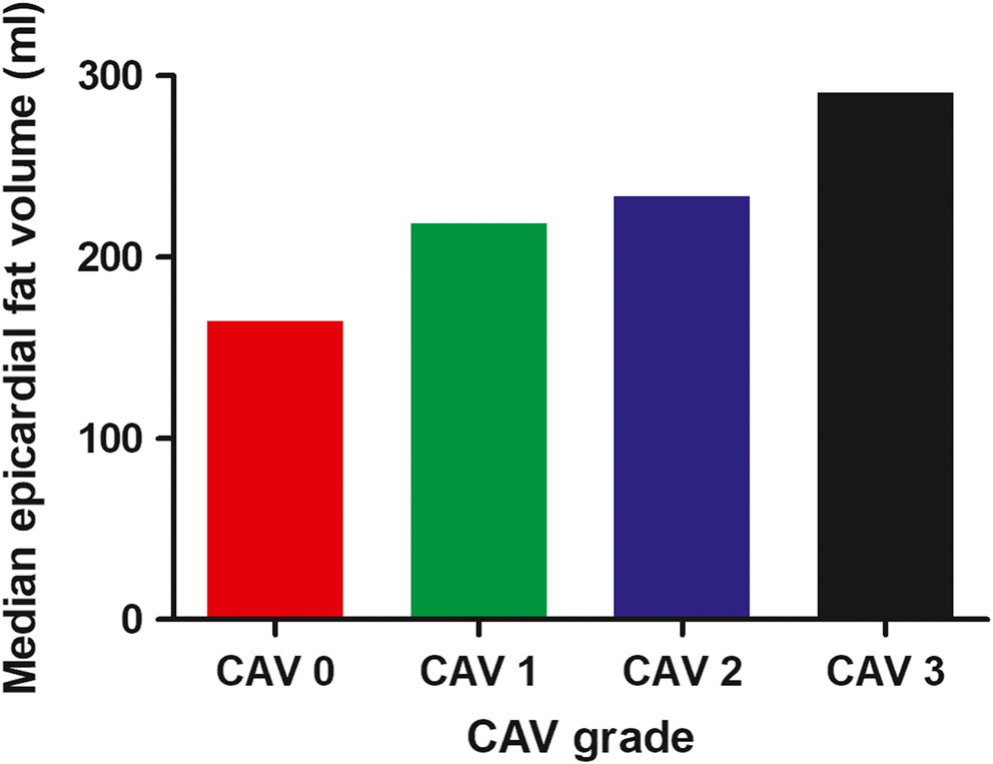
The median epicardial fat volume stratified by cardiac allograft vasculopathy grade (*p < *0.001). Abbreviations: CAV; cardiac allograft vasculopathy

The median EFV on CT was 208.4 (128.9–276.0) mL. The overall prevalence of CAV was 43% of which 32 (22%) had CAV grade 1, 14 (9%) had CAV grade 2, and 18 (12%) had CAV grade 3. When stratified by CAV grade, the EFV ranged from a median of 164.7 to 290.6 mL for CAV grades 0 and 3, respectively (Fig. 2, *p* < 0.001).

### Association of epicardial fat volume with CAV

We found a relationship between larger volumes of epicardial fat (per one unit increase of log-transformed EFV) with higher CAV grades (OR 5.23 (2.47–11.06), *p* < 0.001) (Table 2, model 1). Even after adjustments for sex and age of the recipient and a propensity score including risk factors for CAV, EFV was significantly associated with CAV (model 2: OR 5.17 (2.29–11.69), *p* < = 0.001, model 3: OR 2.73 (1.01–7.41), *p* = 0.049. Additional models are demonstrated in Supplemental Table S1.

**Table 2:** Ordinal regression analysis investigating the association between epicardial fat volume and cardiac allograft vasculopathy

	Cardiac allograft vasculopathy		
	OR (95% CI)		
	Model 1	Model 2	Model 3
Volume of epicardial fat*	5.23 (2.47–11.06)	5.17 (2.29–11.69)	2.73 (1.01–7.41)
*p* value	< 0.001	< 0.001	0.05**

Model 1: Unadjusted

Model 2: Adjusted for sex and age of the recipient

Model 3: Adjusted with a propensity score which included the following parameters: sex and age of the recipient, time since heart transplantation, BMI recipient at the CT scan, diabetes, smoking between heart transplantation and CT scan, number of rejections, CMV infection post-heart transplant, ischemia time

*Ln(epicardial fat volume) – transformed

**p = 0.049

Abbreviations: *BMI*, body mass index; *CI*, confidence interval; *CMV*, cytomegalovirus; *CT*, computed tomography; *EFV*, epicardial fat volume; *OR*, odds ratio

### Determinants of epicardial fat volume

**Fig. 3:**
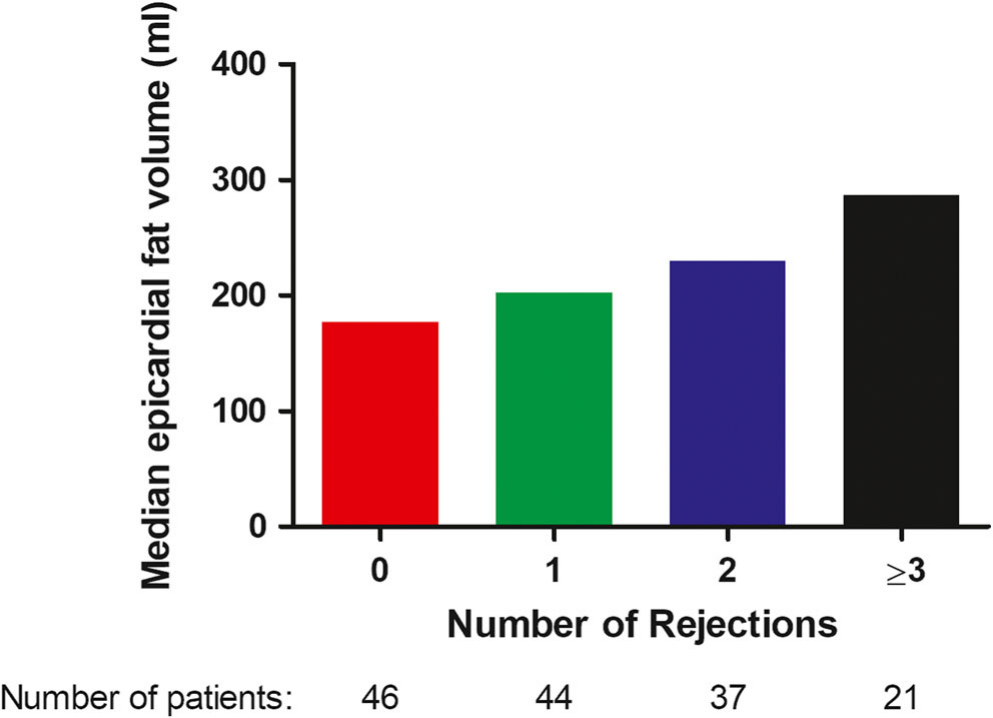
The median epicardial fat volume stratified by the number of rejections post-heart transplantation (*p* = 0.002)

We found independent associations of sex of the recipient (β −0.36 (−0.51; −0.21), *p* < 0.001), the number of rejections (β 0.11 (0.05; 0.18), *p* = 0.001), donor age (β 0.01 (0.00; 0.01), *p* = 0.008), time between HT and CT scan (β 0.02 (0.01; 0.03), *p* = 0.003), BMI of the recipient at the time of the CT (β 0.04 (0.02; 0.05), *p* < 0.001), and diabetes mellitus (β 0.18 (0.03; 0.32), *p* = 0.02) with EFV in multivariable analysis. All the included variables are displayed in Table 3. When specifically investigating the association between EFV and the number of rejections, we found larger volumes of epicardial fat with every rejection episode that occurred (Fig. 3, *p* = 0.002).

**Table 3:** Linear regression analysis to determine risk factors for Ln(epicardial fat volume)

	Univariable	*p* value	Multivariable	*p* value
Sex recipient (female vs male)	− 0.42 (− 0.58; − 0.27)	< 0.001	− 0.36 (− 0.51; − 0.21)	< 0.001
Age recipient	0.01 (0.01; 0.02)	< 0.001	0.00 (− 0.01; 0.01)	0.91
Sex donor (female vs male)	− 0.12 (− 0.29; 0.04)	0.15	− 0.04 (− 0.17; 0.09)	0.53
Age donor	0.01 (0.00; 0.01)	0.02	0.01 (0.00; 0.01)	0.008
Number of rejections *	0.14 (0.07; 0.22)	< 0.001	0.11 (0.05; 0.18)	0.001
Time between HT and CT	0.03 (0.01; 0.04)	< 0.001	0.02 (0.01; 0.03)	0.003
Prednisone use at CT	0.33 (0.16; 0.50)	< 0.001	− 0.01 (− 0.16; 0.14)	0.89
Total Cholesterol	0.12 (0.03; 0.20)	0.007	0.02 (− 0.13; 0.17)	0.81
LDL	0.13 (0.03; 0.23)	0.01	0.10 (− 0.07; 0.27)	0.24
BMI recipient at CT	0.06 (0.04; 0.07)	< 0.001	0.04 (0.02; 0.05)	< 0.001
Diabetes mellitus	0.27 (0.08; 0.45)	0.005	0.18 (0.03; 0.32)	0.02
Smoking post-HT	0.08 (− 0.13; 0.29)	0.43		

*(≥ 3 rejections combined as 1 group)

Regression coefficients of the (univariable and multivariable) linear regression analysis are demonstrated with 95% confidence intervals

Abbreviations: *BMI*, body mass index; *CT*, computed tomography; *HT*, heart transplantation; *LDL*, low-density lipoprotein

**Fig. 4:**
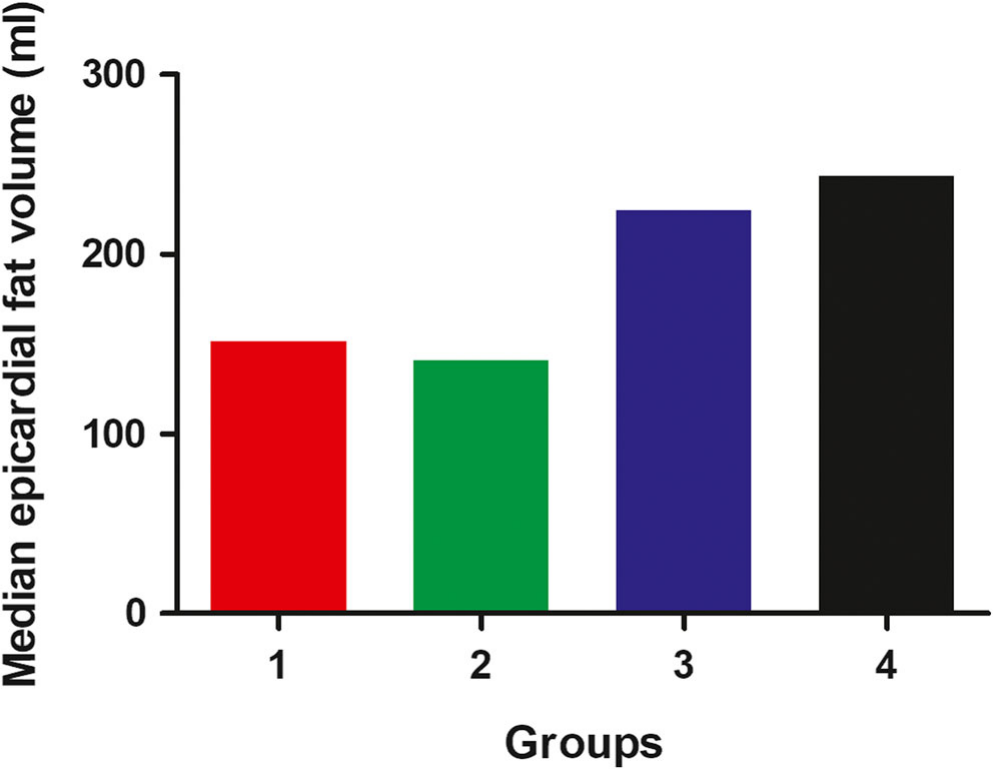
The median epicardial fat volume stratified by recipient/donor sex (*p* < 0.001). Group 1: female recipient, female donor; group 2: female recipient, male donor; group 3:male recipient, female donor; group 4: male recipient, male donor

In the whole study population, 51 (34%) of the male recipient had a male donor, 44 (30%) of the male recipients had a female donor, 13 (9%) female recipients had a male donor, and 41 (28%) of the female recipients had a female donor. When the median EFV of male recipients is compared to the median EFV of female recipients, male recipients have a significantly higher EFV (232.7 mL vs. 147.2 mL respectively, *p* < 0.001) irrespective of donor sex. The distribution of EFV per group is demonstrated in Fig. 4 (*p* < 0.001).

## Discussion

Using a novel CCTA-based strategy for the follow-up of HT patients, we evaluated the amount of epicardial fat as a potential risk factor for CAV. We showed that patients post-HT have high epicardial fat volumes and that larger amounts of epicardial fat were associated with higher CAV grades even after correction for cardiovascular and transplantation-related risk factors. Furthermore, parameters that were related to an increased EFV were: the male sex of the recipient, the number of rejections, the age of the donor, the time between HT and CT scan, the BMI of the recipient, and diabetes mellitus. Male recipients had significantly more EFV compared to female recipients, irrespective of the donor sex.

To our knowledge, this is the first study investigating the association between EFV and CAV. EFV may play an important role in the development of CAV, especially given the near proximity of epicardial fat to the coronary arteries, and its excretion of pro-inflammatory factors [19, 20]. This is further corroborated by recent evidence stating that epicardial fat could play a role in the formation of CAV due to lymphocytes [5, 19]. Lymphoid nodules have been demonstrated in the epicardial fat post-HT [19, 23]. These nodules get organized and attract T-cells. Moreover, epicardial adipocytes secrete more pro-inflammatory (IL-6, IL-8) than anti-inflammatory cytokines (adiponectin), especially in diabetic and obese patients [19]. Further basic science studies are needed to confirm this association in heart transplantation recipients.

In our study, the median EFV was 208.4 (128.9–276.0) mL. When comparing this EFV to large population studies such as the Framingham Heart Study, the Heinz Nixdorf Recall cohort, the EISNER study and the Rotterdam study, the EFV in these studies was significantly lower (mean of 122 mL, 86 mL, 89 mL, and a median of 102 mL, respectively) [10–13]. In the Heinz Nixdorf Recall cohort, the population was divided into quartiles with a mean EFV in the highest quartile of 151 mL [11]. This demonstrates that patients post-HT have a tremendous amount of epicardial fat compared to the general population and could possibly explain why these patients experience CAV early post-HT [1, 2].

EFV was significantly associated with CAV in our study which is in line with results from atherosclerosis studies demonstrating a higher risk of atherosclerosis in patients with higher EFV [10–14, 16]. Risk factors for CAV include not only donor age, recipient sex, acute rejections, and time since HT but also traditional cardiovascular risk factors such as diabetes mellitus, hypertension, and hypercholesterolemia [1, 4, 5, 24]. Some of these factors were associated with EFV in our study. This could clarify how some risk factors are associated with CAV. One example in this perspective is the sex of the recipient. Male recipients are more likely to develop CAV than female recipients [1]. The reason for this is not fully understood but could be explained by neurohormonal differences between sexes. We have demonstrated that male recipients have a higher amount of epicardial fat, irrespective of the sex of the donor, which could explain the increased incidence of CAV in male recipients. The question that still needs to be answered is whether the effects of the risk factors associated with CAV are via epicardial fat or whether these risk factors have an effect on CAV both directly as well as indirectly via epicardial fat. For example, rejection episodes are a known risk factor for CAV [5, 24]. Possibly, rejections of the cardiac allograft can induce adjustments to the endothelium in the coronaries, inducing CAV. Another pathway could be that the EFV is increased due to the inflammatory environment during a rejection episode and/or the high prednisolone dosage that is frequently given to treat acute rejections. It is well known that patients who use prednisolone have an increased amount of body fat [25]. A study investigating the impact of steroid therapy on epicardial fat depositions found that patients with a rheumatic disorder who were on chronic steroid therapy had significantly more EFV than patients without steroid therapy [26]. Moreover, patients with high dosage steroid therapy also had more EFV than patients on low dose steroid therapy [26]. This could at least partially explain the increased amount of epicardial fat in HT recipients and could hint towards the cumulative effect that prednisolone has on EFV (daily dosage as well as the high dosages given during rejection).

Currently, no specific treatment option for CAV is available. Two of the main treatments to date for CAV are statins (recommended for all patients post-HT) and mTOR-inhibitors (everolimus and sirolimus) [2]. Everolimus and sirolimus are mostly given to patients who are longer post-HT in whom early stages of CAV have been identified. Even though mTOR-inhibitors could halt the progression of CAV in some patients, the side effects make it impossible to start these drugs in all patients [27]. Even in patients with CAV, it is sometimes discontinued due to the side effects. The association between EFV and CAV found in this study raises the question of whether EFV could be a new target for drug therapies in order to delay CAV development. Several studies have been performed to reduce EFV in the general population. It has been demonstrated that weight loss with a very-low calorie diet, intensive training session, and even bariatric surgery reduces EFV [28]. It is however questionable whether this can also be applied to HT recipients who sometimes have serious comorbidities [1]. With the introduction of proprotein convertase subtilisin/kexin type 9 (PCSK9) inhibitors, sodium-glucose cotransporters-2 (SGLT2) inhibitors, and glucagon-like peptide-1 receptor (GLP-1) agonists more treatment options are becoming available for patients with cardiovascular disease. In the general population, SGLT2 inhibitors and GLP-1 agonists have demonstrated that they could reduce EFV [29–31], while PCSK9 inhibitors have been proposed as a potential drug to reduce EFV [32, 33]. In a recent study investigating a mouse HT model, the authors found a significant reduction in the amount of inflammation and development of CAV after treatment with a GLP-1 agonist [34]. Even though the authors did not investigate the EFV in these mice, the reduction in inflammation (and possibly the development of CAV) could be explained by the reduction in EFV. Recently, several case series and small cohort studies have examined the safety of the abovementioned drugs in the HT population [35, 36]. Studies are now needed to confirm that EFV can be reduced in HT patients and whether this could slow down CAV progression.

Our study has several limitations. Our study is a single-center study which comes with its limitations, even though the study sample was not small. Furthermore, most patients in our population were Caucasian, so whether our results can be extrapolated to other ethnicities is uncertain. Patients with a contrast allergy or an impaired renal function (eGFR < 30 mL/min/1.73 m^2^) had no CCTA (but stress scintigraphy) for CAV screening which could alter our results. No CCTA was performed shortly after HT which is why a comparison with baseline measurements is not possible. However, it is to be expected that the baseline EFVs are comparable or even lower compared to general population studies such as the Framingham and the Rotterdam Study [12, 13], which makes our results even more interesting. We were not able to correct for all risk factors related to CAV, such as donor age, hypertension, and hypercholesterolemia due to the fact that these were related too highly related to other factors or too prevalent in the study population. Furthermore, it was not possible to correct the calcium score, as in patients with a stent, the calcium score could not be determined. This was the case in a significant number of patients with a higher CAV grade. Lastly, CCTA has limitations to describe the CAV grade. The CAV grade classification is based on angiographic findings [4]. In contrast to angiography findings, significant stenosis on CCTA is a stenosis > 50% instead of 70% (Supplemental Material). This could lead to an overestimation of the CAV grade in this study. On the other hand, patients can have abnormalities in the coronary wall, not protruding into the lumen, leading to a CAV grade 0 based on the guidelines, even though abnormalities are seen [8]. Additional studies need to be performed to explore whether patients with a high EFV also have an increased risk to have CAV progression. This could help identify high-risk patients who need more close monitoring and more aggressive treatment of their comorbidities to slow down CAV progression.

In conclusion, we demonstrated that the EFV is significantly increased in HT recipients compared to the general population (even more in male recipients). Larger volumes of epicardial fat were significantly associated with higher CAV grades. Parameters associated with an increased EFV include the sex of the recipient, donor age, rejections, time since HT, the BMI of the recipient, and diabetes mellitus. Further studies are needed to investigate whether the reduction of EFV could slow down the progression of CAV, improving quality of life and survival.

## Supplementary information

ESM 1 (DOCX 23 kb)

## Abbreviations

CAD: Coronary artery disease
CAV: Cardiac allograft vasculopathy
CCTA: Coronary computed tomography angiography
CMV: Cytomegalovirus
EFV: Epicardial fat volume
GLP-1: Glucagon-like peptide-1 receptor
HT: Heart transplantation
HU: Hounsfield unit
ISHLT: International Society for Heart and Lung Transplantation
PCSK9: Proprotein convertase subtilisin/kexin type 9
SCCT: Society for Cardiovascular Computed Tomography
SGLT2: Sodium-glucose cotransporters-2

## Declarations

### Guarantor

The scientific guarantor of this publication is Olivier C. Manintveld.

### Statistics and biometry

One of the authors has significant statistical expertise.

### Study subjects or cohorts overlap

Some study subjects or cohorts have been previously reported in:

Nous FMA, Roest S, van Dijkman ED et al (2021) Clinical implementation of coronary computed tomography angiography for routine detection of cardiac allograft vasculopathy in heart transplant patients. Transpl Int

And:

Budde RPJ, Nous FMA, Roest S et al (2021) CT-derived fractional flow reserve (FFRct) for functional coronary artery evaluation in the follow-up of patients after heart transplantation. Eur Radiol.

However, both studies had a different study population than this study (this study is larger in size) and these articles were written for a different research question/purpose.

### Methodology

• retrospective

• cross-sectional study

• performed at one institution
