# Supplementary material for: Epicardial fat volume is related to the degree of cardiac allograft vasculopathy
Source: Eur Radiol. 2022 Aug 20;33(1):330–8. doi: 10.1007/s00330-022-09029-2 (PMC9755093; doi:10.1007/s00330-022-09029-2)
Supplement: Supplementary file 1 — (DOCX 23 kb) [file 330_2022_9029_MOESM1_ESM.docx]

**Epicardial fat volume is related to the degree of cardiac allograft vasculopathy**

**Supplemental Material and Methods**

**Definitions**

Hypertension was defined as patients who used antihypertensive medication at the time of the CT scan. Significant rejections were defined as rejections which were graded ≥2R according to the ISHLT guidelines(1) or when a patient was treated due to the clinical suspicion of an acute rejection, where a biopsy could not be performed due to an unstable haemodynamic status. Given the low number of patients with ≥ three rejections, these were combined into one group. A cytomegalovirus (CMV) infection was defined when a patient had a replication rate >1000 copies or when a patient experienced symptoms related to a CMV infection.

CAV grade 0 was defined as no plaques protruding the coronary lumen. CAV grade 1 was defined as a stenosis <50% in the primary vessels or any branch stenosis <50%. CAV grade 2 was defined as a stenosis in a single primary vessel ≥50% or isolated branch stenosis ≥50% in two systems. CAV grade 3 was defined as a left main stenosis ≥50%, a stenosis in two or more primary vessels ≥50% or isolated branch stenosis ≥50% in all three systems. Whenever a stent was present that had been implanted previously to treat a significant coronary stenosis, this was regarded as a significant stenosis in that vessel (making patients at least CAV grade 2).

**References**

1. Stewart S, Winters GL, Fishbein MC, et al.: Revision of the 1990 working formulation for the standardization of nomenclature in the diagnosis of heart rejection. J Heart Lung Transplant 2005;24:1710-20.

|  |  |  | Cardiac Allograft Vasculopathy | | | |  |
| --- | --- | --- | --- | --- | --- | --- | --- |
|  |  |  | OR (95% CI) | | | |  |
|  | *Model 1* | | | *Model 2* | *Model 3* | *Model 4* | |
| Volume of epicardial fat* | 3.22 (1.38-7.47) | | | 3.47 (1.30-9.30) | 2.69 (1.11-6.52) | 2.90 (1.04-8.07) | |
| P-value | 0.007 | | | 0.01 | 0.028 | 0.041 | |

**Supplemental Table S1. Additional ordinal regression analysis investigating association epicardial fat volume and cardiac allograft vasculopathy**

Model 1: Adjusted for recipient age at HT, the sex of the recipient and time since HT.

Model 2: As Model 1, and additionally adjusted for BMI of the recipient, smoking between HT and CCTA, and diabetes mellitus.

Model 3: As Model 1, and additionally adjusted for number of rejections.

Model 4: Adjusted for all variables in Model 1, 2 and 3

* Ln(epicardial fat volume) – transformed

Abbreviations: EFV, epicardial fat volume; OR, odds ratio; CI, confidence interval.
